# S100A8/A9 is not essential for the development of inflammation and joint pathology in interleukin-1 receptor antagonist knockout mice

**DOI:** 10.1186/s13075-021-02602-y

**Published:** 2021-08-19

**Authors:** Irene Di Ceglie, Peter L. E. M. van Lent, Edwin J. W. Geven, Marije I. Koenders, Arjen B. Blom, Thomas Vogl, Johannes Roth, Martijn H. J. van den Bosch

**Affiliations:** 1 Experimental Rheumatology, Radboud University Medical Center, Geert Grooteplein 28, 6525 GA Nijmegen, the Netherlands; 2 Institute of Immunology, University of Münster, Münster, Germany

**Keywords:** IL-1 receptor antagonist, Cartilage erosion, Bone erosion, Myeloid cells, S100A8/A9

## Abstract

**Background:**

Excessive osteoclast activity, which is strongly stimulated by pro-inflammatory mediators, results in bone and cartilage degeneration as central features of many arthritides. Levels of the alarmin S100A8/A9 and interleukin (IL)-1β are both increased in arthritis patients and correlate with disease activity and progression of tissue erosion. We previously presented S100A8/A9 as a good biomarker for joint inflammation and arthritis pathology under circumstances of high IL-1 signaling in mice that lack the gene encoding IL-1 receptor antagonist (*Il1rn*^*−/−*^ mice). Here, we investigated whether S100A8/A9 is also actively involved in the development of joint inflammation and both cartilage and bone pathology under these conditions by comparing *Il1rn*^*−/−*^ mice with mice that have an additional deficiency for *S100a9* (*Il1rn*^*−/−*^X*S100a9*^*−/−*^).

**Methods:**

*Il1rn*^*−/−*^X*S100a9*^*−/−*^ on a BALB/c background were obtained by crossing *S100a9*^*−/−*^ mice and *Il1rn*^*−/−*^ mice. Arthritis incidence and severity were macroscopically scored. Myeloid cell populations in the bone marrow and spleen were determined using flow cytometry. In vitro osteoclastogenesis of bone marrow cells was evaluated with TRAP staining. Microscopic joint inflammation, cartilage degeneration, and bone destruction were evaluated using histology of ankle joints of 12- and 20-week-old mice.

**Results:**

Macroscopically scored arthritis severity was comparable between *Il1rn*^*−/−*^ and *Il1rn*^*−/−*^X*S100a9*^*−/−*^ mice. Inflammation, cartilage erosion, and bone erosion were clearly present in 12-week-old mice of both strains lacking *Il1rn*^*−/−*^, but not significantly different between *Il1rn*^*−/−*^X*S100a9*^*−/−*^ and *Il1rn*^*−/−*^. Moreover, we observed that the numbers of neutrophils and monocytes were increased by the absence of *Il1rn*, which was affected by the absence of *S100a9* only in the spleen but not in the bone marrow*.* In line with our other findings, the absence of *S100a9* did not affect the osteoclastogenic potential of osteoclast precursors in the absence of *Il1rn*. Finally, in agreement with the findings in early arthritis development in 12-week-old mice, cartilage and bone erosion in 20-week-old mice was significantly higher in both *Il1rn*^*−/−*^ strains, but the additional absence of *S100a9* did not further affect tissue pathology.

**Conclusion:**

S100A8/A9 deficiency does not significantly affect inflammation and joint destruction in mice with high IL1β signaling suggesting that S100A8/A9 is not essential for the development of arthritis under these conditions.

**Supplementary Information:**

The online version contains supplementary material available at 10.1186/s13075-021-02602-y.

## Introduction

Arthritides form a large and heterogeneous group of diseases and predominantly affect articular joints. Erosion of the articular cartilage and of the peri-articular bone are central features in most of these diseases and are mediated by osteoclasts, multinucleated cells that differentiate from myeloid precursors under the influence of macrophage colony-stimulating factor (M-CSF) and receptor activator of nuclear factor kappa-Β ligand (RANKL) [1, 2]. During steady-state conditions, balanced bone resorption by osteoclasts and bone formation by osteoblasts ensure homeostatic bone turnover and remodeling. Under arthritic conditions, however, the excess of immune cells present in the synovium of affected joints produce a plethora of damage-associated molecular patterns (DAMPs), also referred to as alarmins, and pro-inflammatory factors that strongly promote the differentiation and resorptive activity of osteoclasts, leading to excessive bone erosion [3–6].

Two key factors that are highly produced under arthritic conditions are the alarmin S100A8/A9 and interleukin (IL)-1β. Elevated levels of S100A8/A9 can be measured in the synovium, synovial fluid, and serum during various rheumatic diseases, and these levels correlate with disease activity and radiographic progression, suggesting its involvement in pathology [7–17]. More specifically, we have previously shown that S100A8/A9 promotes osteoclast-mediated bone resorption [6]. This alarmin is present in the cytoplasm of myeloid cells, but upon cell stress as the result of e.g. inflammation or tissue damage, S100A8/A9 is secreted whereupon it rapidly activates the immune system, predominantly via binding to Toll-like receptor 4 [18–20]. High IL-1 signaling has been found in several types of seronegative arthritis and is most pronounced in systemic onset juvenile idiopathic arthritis [21–23]. IL-1β can bind to the type 1 IL-1 receptor (IL-1RI) or the non-signaling type 2 IL-1 receptor (IL-1RII). The IL-1 receptor antagonist (IL-1RA) competitively blocks the binding of IL-1β to IL-1RI and thereby decreases IL-1 signaling [24]. Mice that are deficient in the gene encoding IL-1RA (*Il1rn*^*−/−*^ mice) therefore represent an ideal model to study high IL-1 signaling. These mice spontaneously develop non-immune complex-mediated joint pathology which includes severe joint inflammation, cartilage destruction, and bone erosion that is associated with marked osteoclast activity [25].

A previous study from our group showed that the serum levels of S100A8/A9 were a potent biomarker for joint inflammation and destruction in these *Il1rn*^*−/−*^ mice. Serum levels of S100A8/A9 were markedly increased in these mice compared to wild type controls and strongly correlated with various disease parameters, such as macroscopic arthritis severity and histologically scored inflammation, bone erosion, chondrocyte death, proteoglycan depletion, and (PG) cartilage erosion. Moreover, we demonstrated that serum S100A8/A9 levels early after disease onset were prognostic for the disease outcome [26]. Therefore, in the present study, we investigated the functional role of S100A8/A9 in the development of joint inflammation and destruction of cartilage and bone under conditions of high IL-1 signaling. Hereto, we generated *Il1rn*^*−/−*^X*S100a9*^*−/−*^ double knockout mice and compared various joint pathology parameters between these double knockout and *Il1rn*^*−/−*^ mice, whereas *S100a9*^*−/−*^ and wild type (WT) mice were used as control. Moreover, we investigated various immune cell subsets in the bone marrow and spleen of these mice and compared the osteoclastogenic potential of osteoclast precursors between the various strains.

## Materials and methods

### Mice

*Il1rn*^*−/−*^X*S100a9*^*−/−*^ on a BALB/c background were obtained by crossing *S100a9*^*−/−*^ mice (*own facilities*) and *Il1rn*^*−/−*^ (previously kindly provided by Dr. M. Nicklin, The University of Sheffield, Sheffield, UK) [27]. Single knockout *Il1rn*^*−/−*^ and *S100a9*^*−/−*^ control strains were then generated by crossing the heterozygous *Il1rn*^*+/−*^X*S100a9*^*+/−*^. BALB/c wild type (WT) controls were purchased from Envigo. Development of arthritis in the ankle and paws was macroscopically scored on a scale from 0 to 2 per paw (0, no redness and swelling; 0.25, slight redness; 0.5, slight redness and swelling; 0.75–1, mild redness and swelling; 1.25–1.5, moderate redness and swelling; 1.75–2, severe redness and swelling) and the arthritis severity is presented as the area under the curve (AUC). Mice were sacrificed at the age of 12 weeks or 20 weeks old. Mice were housed under standard housing conditions: filter top cages, 12 h light-dark cycle, and ad libitum access to animal chow and water. All experiments involving animals were conducted according to the Dutch law and approved by the Dutch Central Authority for Scientific Procedures on Animals (#AVD103002015115).

### Histological analysis

Total ankle joints were isolated from 12- and 20-week-old mice, fixed in 4% phosphate-buffered formalin at room temperature, decalcified in 10% ethylenediaminetetraacetic acid (EDTA) at 4°C for 2 weeks, embedded in paraffin, and 7-μm coronal sections were prepared, representing the entire depth of the joint. Sections were stained with hematoxylin and eosin and Safranin-O and Fast Green for histological analysis. Inflammation of the entire ankle joint was arbitrarily scored on a scale from 0 (no inflammation) to 3 (severe inflammation). Bone destruction was evaluated in 8 well-defined areas along the tibia, talus, and navicular with a score ranging from 0 (no erosion) to 3 (connection between the joint cavity and bone marrow or more extensive bone destruction). Degeneration of the articular cartilage layers at the tibia, talus, and navicular bone were scored on a scale from 0 (no erosion) to 3 (complete erosion of the calcified cartilage layer). Proteoglycan depletion in the articular cartilage was evaluated in the same four locations as cartilage erosion and scored on a scale from 0 (no PG depletion) to 3 (complete PG depletion). A total of 3 sections per joint from various depths were scored and results from all locations were averaged (Additional file 1).

### Flow cytometric analysis

Total bone marrow cells from femurs of 12-week-old mice were isolated by crushing the femurs of mice with mortar and pestle in medium and passing the cell suspension through a 70-μm nylon cell strainer. Total cell populations of whole spleen cells were isolated by mashing the spleen over a 70-μm nylon cell strainer. After lysis of erythrocytes in lysis buffer (155 mM NH_4_Cl 12 mM KHCO_3_ 0,1 mM EDTA pH 7.3), cells were counted and subsequently incubated with Fc-blocking antibody (BD Pharmingen anti-mouse CD16/CD32, BD Biosciences). Cells were then stained with the following mix of antibodies: CD11b-fluorescein isothiocyanate (FITC), CD3-phycoerythrin (PE), Ter-119-PE, CD45R/B220-PE, CD49b-PE, NK1.1-PE, Ly6G-allophycocyanin (APC), Ly6C-allophycocyanin-cyanine 7 (APC-Cy7), and an eFluor450 fixable viability dye. Samples were acquired with a Gallios flow cytometer (Beckman Coulter Life Sciences) and data analysis was performed with Kaluza Analysis Software 2.1 (Beckman Coulter Life Sciences). The gating strategy is shown in Additional file 2.

### Bone marrow-derived osteoclast differentiation

Total bone marrow cells were isolated from the femurs of 12-week-old mice and were seeded in Petri dishes at a density of 10^6^ cells/mL in 10 mL of α-minimum essential medium (αMEM) (Thermo Fisher Scientific), supplemented with 10% fetal calf serum (FCS), 100 U/mL penicillin, and 100 μg/mL streptomycin in the presence of 30 ng/mL recombinant mouse (rm)M-CSF (R&D Systems). At day 3 of culture, cells were trypsinized and seeded in 96-well plates at a density of 25 × 10^4^ cells/well in αMEM supplemented with 30 ng/mL of rmM-CSF and 20 ng/mL rmRANKL (R&D Systems). The culture medium was refreshed after 3 days. After 4 days of differentiation in the presence of RANKL, cells were fixed with 4% PFA and stained for tartrate-resistant acid phosphatase (TRAP), using the Leukocyte Acid Phosphatase Kit (Sigma-Aldrich) according to the manufacturer’s protocol. Cells with three or more nuclei were considered osteoclasts and were further scored as small osteoclasts with 3–5 nuclei and bigger osteoclasts with ≥6 nuclei.

### Statistical analysis

Statistical differences between multiple groups were calculated using a Kruskal-Wallis test followed by Dunn’s multiple comparison test. A two-way ANOVA was performed to study the effects of *Il1rn* and *S100a9* deficiencies and their interaction. Statistical differences between two groups were tested using a Mann-Whitney test. Correlation between parameters was determined by calculating Spearman’s rank correlation coefficients. All analyses were performed using Graph Pad Prism 5.03 (Graph Pad Software) and *P*-values less than 0.05 were considered significant.

## Results

### The absence of S100a9 does not affect arthritis severity in *Il1rn*^*−/−*^ mice during early arthritis

**Fig. 1:**
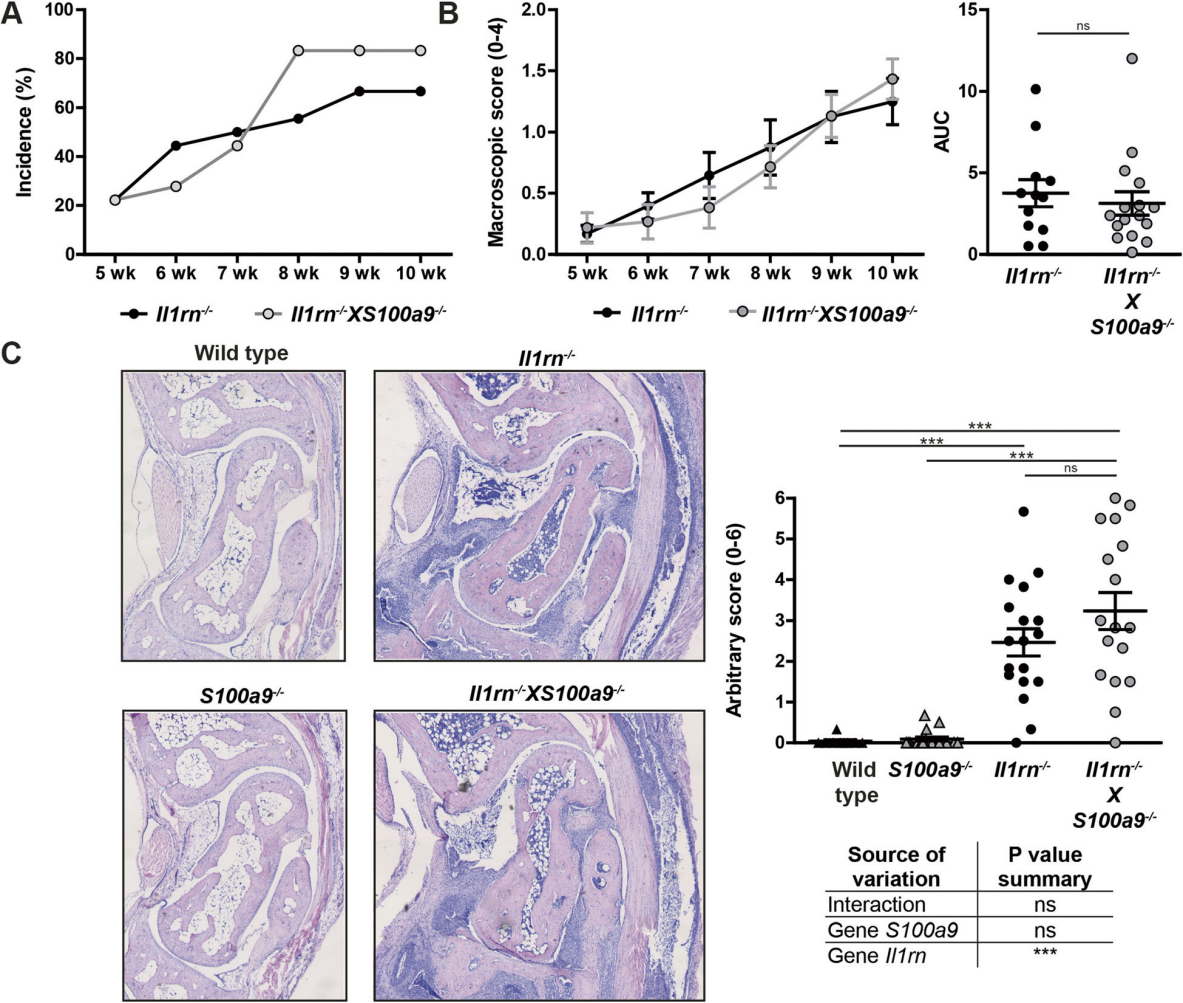
The absence of s100a9 does not affect inflammation in the ankle joints of *Il1rn*^*−/−*^ mice. Arthritis incidence, based on the macroscopic score of the ankle joints, was increased in *Il1rn*^*−/−*^X*S100a9*^*−/−*^ compared to *Il1rn*^*−/−*^ mice (**A**). However, the severity of arthritis in the positive mice was comparable (**B**). Representative photomicrographs of hematoxylin and eosin-stained sections showing examples of inflammation in the ankle joints of 12-week-old wild type (WT), *S100a9*^*−/−*^, *Il1rn*^*−/−*^, and *Il1rn*^*−/−*^X*S100a9*^*−/−*^ mice (**C**, original magnification ×50). Quantification of the degree of inflammation of the ankle joints using an arbitrary score showed that the strains deficient in *Il1rn* developed significantly more inflammation compared to the *S100a9*^*−/−*^ and WT controls*.* However, no significant differences in inflammation were observed between *Il1rn*^*−/−*^X*S100a9*^*−/−*^ and *Il1rn*^*−/−*^ mice. Line graphs are shown, representing the percentage of positive mice in **A** and mean ± SEM values of arthritis severity in **B**. Scatterplots are shown, with horizontal and vertical lines representing mean ± SEM values (**C**). Each data point represents the sum score of the right and left ankle joint of one mouse. ns not significant, ***P < 0.001. Results of the two-way ANOVA with interaction are presented in the table

First, we determined whether, in line with previous observations from our lab, serum S100A8/A9 levels correlated with histologic inflammation in *Il1rn*^*−/−*^ mice and we confirmed a strong correlation in the present set of mice **(**Additional file 3A). Next, we investigated whether differences in the incidence and macroscopic severity of arthritis could be observed between 12-week-old *Il1rn*^*−/−*^ and *Il1rn*^*−/−*^ mice that additionally lacked *S100a9*. We observed a slightly increased disease incidence in *Il1rn*^*−/−*^X*S100a9*^*−/−*^ as compared to *Il1rn*^*−/−*^ mice (Fig. 1A). However, the course of arthritis severity development in the positive mice unexpectedly showed no significant differences between the two strains in the cumulative score, represented by the area under the curve (Fig. 1B). As expected, *S100a9*^*−/−*^ and WT controls did not develop any macroscopically visible arthritis. In agreement with these findings, microscopic scoring of tissue sections for inflammation in the ankle joints showed that the strains that were deficient in *Il1rn* developed significantly more inflammation compared to the *S100a9*^*−/−*^ and WT controls, whereas no significant differences could be observed between *Il1rn*^*−/−*^X*S100a9*^*−/−*^ and *Il1rn*^*−/−*^ mice (Fig. 1C).

### S100A8/A9 is not functionally involved in cartilage and bone erosion in *Il1rn*^*−/−*^ mice during early arthritis

**Fig. 2:**
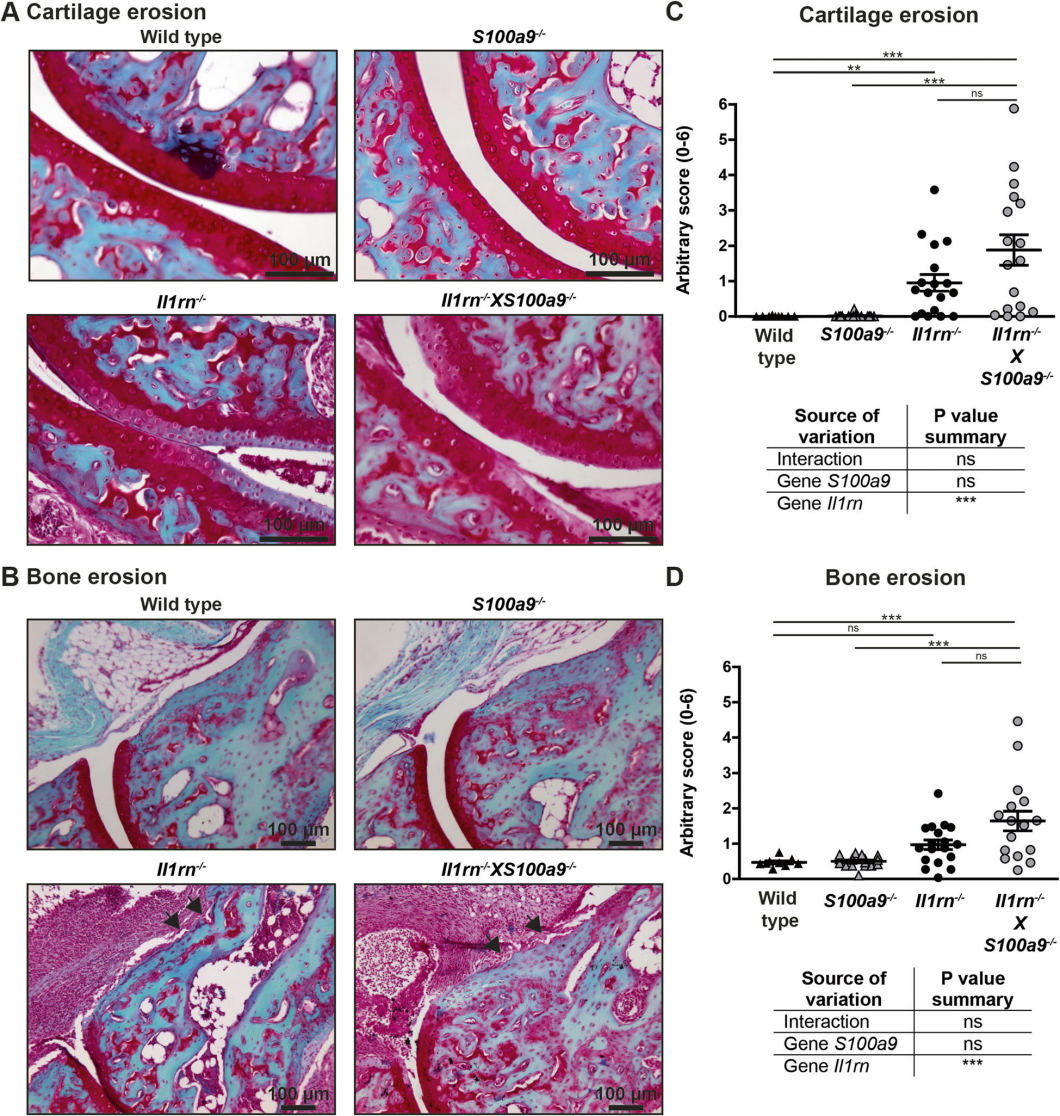
The absence of S100A9 does not ameliorate cartilage and bone erosion in 12-week-old *Il1rn*^*−/−*^ *mice.* Representative photomicrographs of Safranin-O/Fast Green-stained sections showing cartilage and bone erosion in the ankle joints of wild type, *S100a9*^*−/−*^, *Il1rn*^*−/−*^, and *Il1rn*^*−/−*^X*S100a9*^*−/−*^ mice (**A**, original magnification ×200 and **B**, original magnification ×100). Quantification of cartilage erosion at the tibia, proximal and distal sides of the talus, and proximal side of the navicular bone with an arbitrary score showed increased cartilage erosion in *Il1rn-*deficient strains compared to wild type and *S100a9*^*−/−*^ mice. However, no significant differences were observed between *Il1rn*^*−/−*^X*S100a9*^*−/−*^ and *Il1rn*^*−/−*^ mice (**C**). Additionally, quantification of bone erosion at 8 locations along the tibia, talus, and navicular bones with an arbitrary score showed increased bone erosion in *Il1rn-*deficient strains compared to wild type and *S100a9*^*−/−*^ mice while no significant differences were observed between *Il1rn*^*−/−*^X*S100a9*^*−/−*^ and *Il1rn*^*−/−*^ mice (**D**). Scatterplots are shown, with horizontal and vertical lines representing mean ± SEM values. Each data point represents the sum score of the right and left ankle joints of one mouse. ns not significant, **P < 0.01, ***P < 0.001. Results of the two-way ANOVA with interaction are presented in the table

Next, we determined whether the absence of *S100a9* affected cartilage degeneration and bone erosion in 12-week-old mice. Again in agreement with our previous observations, serum S100A8/A9 levels in the *Il1rn*^*−/−*^ mice used here strongly correlated with both cartilage and bone erosion (Additional file 3B and 3C). As presented in Fig. 2A and B, WT and *S100a9*^*−/−*^ mice hardly had any degeneration of the articular cartilage or bone erosion, whereas *Il1rn*^*−/−*^ and *Il1rn*^*−/−*^X*S100a9*^*−/−*^ mice showed severe pathology. Quantification showed that both cartilage erosion (Fig. 2C) and bone erosion (Fig. 2D) were significantly higher in both strains lacking *Il1rn*^*−/−*^ as compared to mice that were sufficient in *Il1rn*. In the strains deficient in *Il1rn*, the number of mice with severe pathology seemed highest in the *Il1rn*^*−/−*^X*S100a9*^*−/−*^ group, where particularly the distal part of the talus and the navicular bone (data not shown) showed severe erosions of both bone and cartilage. However, for both parameters, this did not result in a significant increase compared to *Il1rn*^*−/−*^ mice.

### S100A8/A9 levels affect the composition of myeloid cell populations in the spleen but not in the bone marrow of *Il1rn*^*−/−*^ mice

**Fig. 3:**
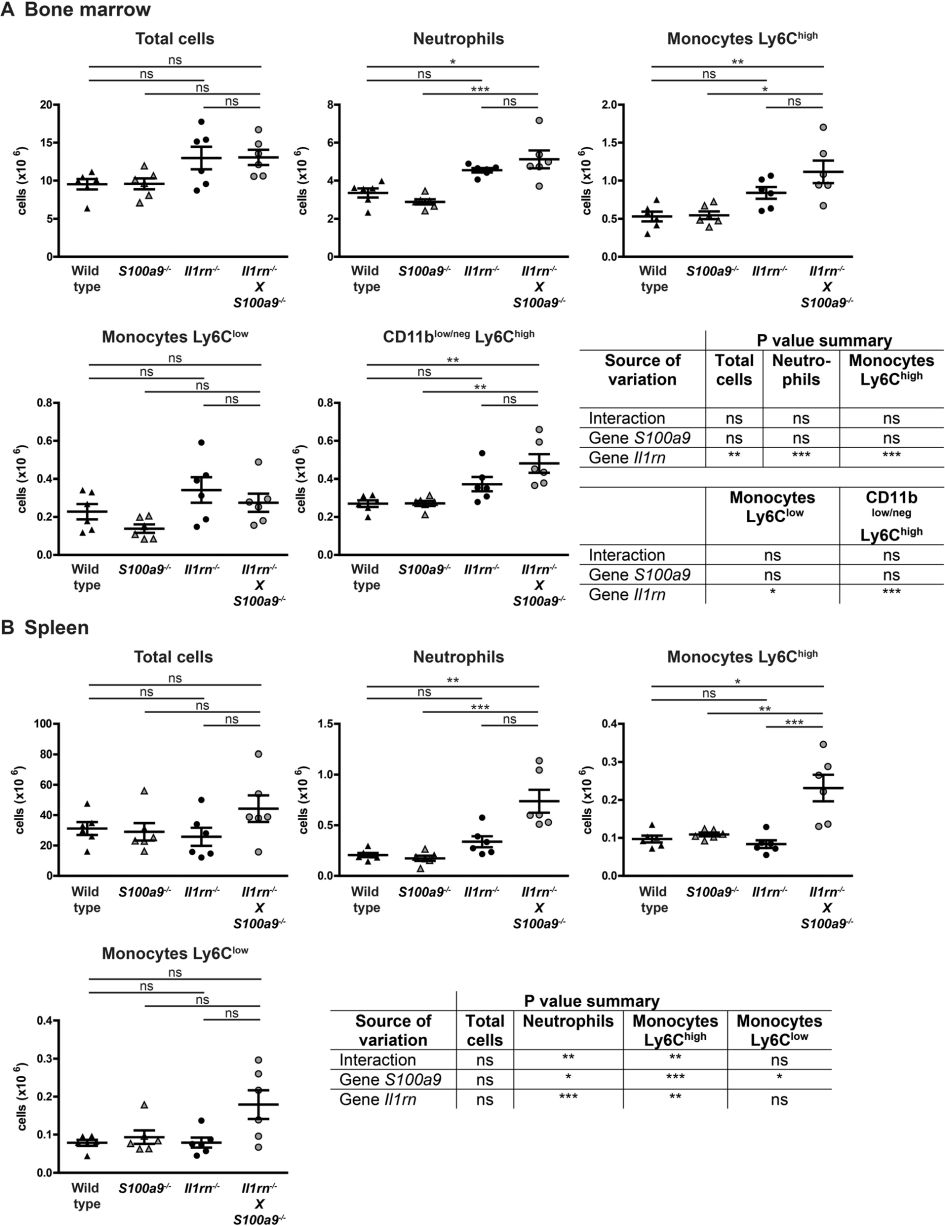
The absence of S100A9 affects the composition of myeloid populations in the spleen of *Il1rn*^*−/−*^ mice. Total cell counts in the bone marrow were significantly higher in mice deficient in *Il1rn*, but not different between *Il1rn*^*−/−*^ and *Il1rn*^*−/−*^X*S100a9*^*−/−*^ mice. Flow cytometry analysis showed that the number of CD11b^+^Ly6G^high^ neutrophils, pro-inflammatory CD11b^+^Ly6G^−^Ly6C^high^ monocytes and the more immunomodulatory CD11b^+^Ly6G^−^Ly6C^low^ monocytes, and CD11b^neg/low^Ly6G^−^Ly6C^high^ osteoclast progenitors were significantly increased in the absence of *Il1rn.* However, no significant differences were observed for any of these populations between *Il1rn*^*−/−*^X*S100a9*^*−/−*^ and *Il1rn*^*−/−*^ mice (**A**). In contrast to the bone marrow, total cell numbers were not increased in the spleen of *Il1rn*-deficient mouse strains. Additionally, an interaction between the absence of *Il1rn* and *S100a9* was present for the numbers of neutrophils and Ly6C^high^ monocytes but not for the Ly6C^low^ monocytes (**B**). Scatterplots are shown, with horizontal and vertical lines representing mean ± SEM values. *P < 0.05, **P < 0.01, ***P < 0.001. Results of the two-way ANOVA with interaction are presented in the table

In the next set of experiments, we set out to determine whether the absence of *S100a9* in *Il1rn*^*−/−*^ mice affected the composition of various myeloid cell populations in the bone marrow and spleen. These cells are progenitors of among others osteoclasts, which are key cells in the erosion of bone and cartilage in these mice. Total cell counts in the bone marrow were significantly higher in mice that were deficient in *Il1rn* compared to the control strains, but not different between *Il1rn*^*−/−*^ and *Il1rn*^*−/−*^X*S100a9*^*−/−*^ mice (Fig. 3A). Next, we determined the number of CD11b^+^Ly6G^high^ neutrophils, pro-inflammatory CD11b^+^Ly6G^−^Ly6C^high^ monocytes and the more immunomodulatory CD11b^+^Ly6G^−^Ly6C^low^ monocytes, and CD11b^neg/low^Ly6C^high^ osteoclast progenitors. Cell numbers in all these populations were significantly increased in the absence of *Il1rn.* Although a trend towards increased numbers of neutrophils, Ly6C^high^ monocytes, and CD11b^neg/low^Ly6C^high^ osteoclast progenitors and a negative trend for Ly6C^low^ monocytes could be observed in the *Il1rn*^*−/−*^X*S100a9*^*−/−*^ mice compared to *Il1rn*^*−/−*^ mice, this did not reach significance for any of these populations.

In contrast to the bone marrow, total cell numbers in the spleen were unchanged in *Il1rn*-deficient mouse strains (Fig. 3B). Moreover, we observed an interaction effect between the absence of *Il1rn* and *S100a9* for the numbers of neutrophils and Ly6C^high^ but not for the Ly6C^low^ monocytes, which was most likely the result of increased numbers of these cells in the *Il1rn*^*−/−*^X*S100a9*^*−/−*^ mice.

### *Il1rn*^*−/−*^X*S100a9*^*−/−*^ and *Il1rn*^*−/−*^ progenitor cells have a comparable osteoclastogenic potential in vitro

**Fig. 4:**
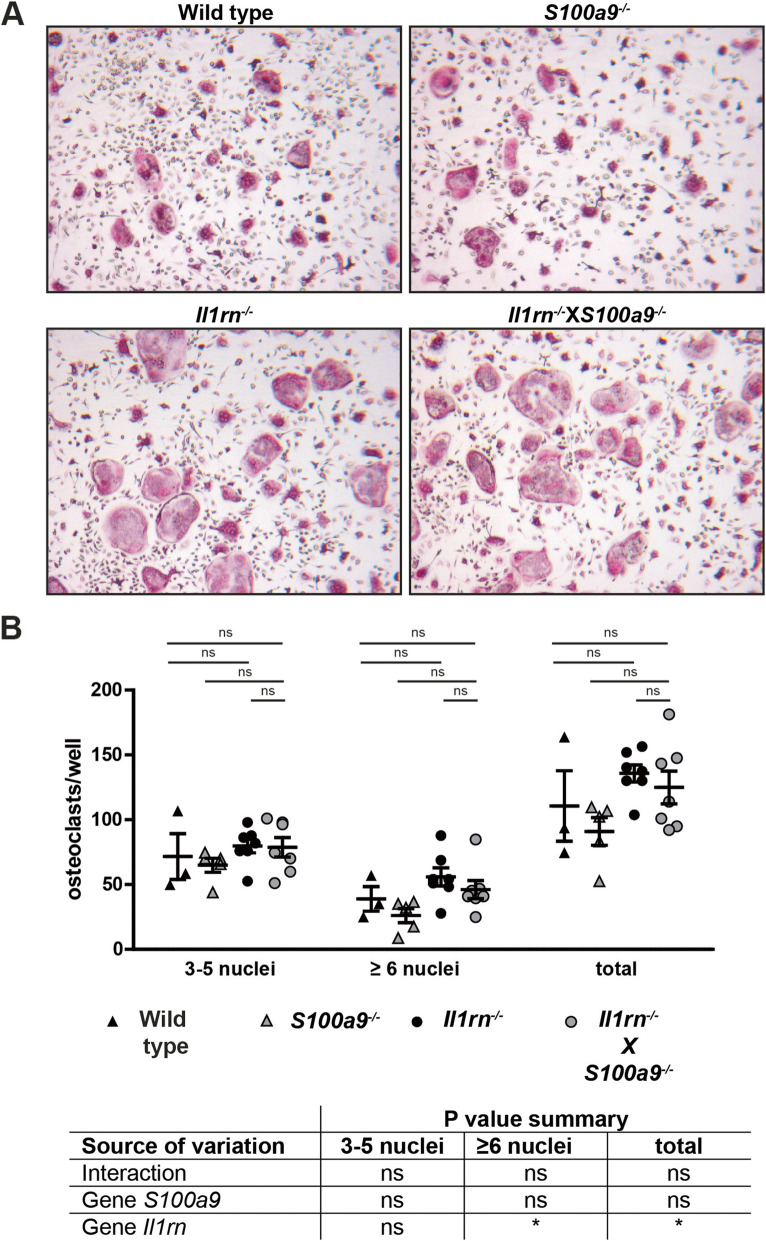
*Il1rn*^*−/−*^X*S100a9*^*−/−*^ and *Il1rn*^*−/−*^ osteoclast precursors have a comparable osteoclastogenic potential in vitro. Photomicrographs of tartrate-resistant acid phosphatase staining of in vitro differentiated osteoclasts derived from bone marrow cells of wild type (WT), *S100a9*^*−/−*^, *Il1rn*^*−/−*^, and *Il1rn*^*−/−*^X*S100a9*^*−/−*^ mice (**A**, original magnification ×40). Quantification showed an increased formation of big (6 or more nuclei) osteoclasts in *Il1rn*-deficient strains compared to *S100a9*^*−/−*^ and WT control cells. No differences were present between *Il1rn*^*−/−*^ and *Il1rn*^*−/−*^X*S100a9*^*−/−*^ cells (**B**). Scatterplots are shown, with horizontal and vertical lines representing mean ± SEM values. *P < 0.01. Results of the two-way ANOVA with interaction are presented in the table

Since osteoclasts are crucial for the bone and cartilage destruction in this experimental arthritis model, we investigated the osteoclastogenic potential of bone marrow cells obtained from all four mouse strains. To adjust for the numbers of osteoclast precursors, we first seeded total bone marrow cells on Petri dishes, stimulated these with GM-CSF, and transferred comparable cell numbers of all strains for the final osteoclast differentiation with RANKL. Bone marrow cells of all strains gave rise to TRAP-positive multinucleated osteoclasts (Fig. 4A). Quantification of the generated osteoclasts showed no major differences in the osteoclastogenic potential of the cells with the four genotypes. Whereas cells from both strains lacking *Il1rn* had more formation of larger osteoclasts (6 nuclei or more) as compared to cells obtained from WT and *S100a9*^*−/−*^ mice, no significant difference could be observed between *Il1rn*^*−/−*^X*S100a9*^*−/−*^ and *Il1rn*^*−/−*^ cells (Fig. 4B).

### The absence of S100A8/A9 does not affect cartilage proteoglycan depletion, cartilage erosion, and bone erosion in ankle joints of *Il-1rn*^*−/−*^ during late arthritis

**Fig. 5:**
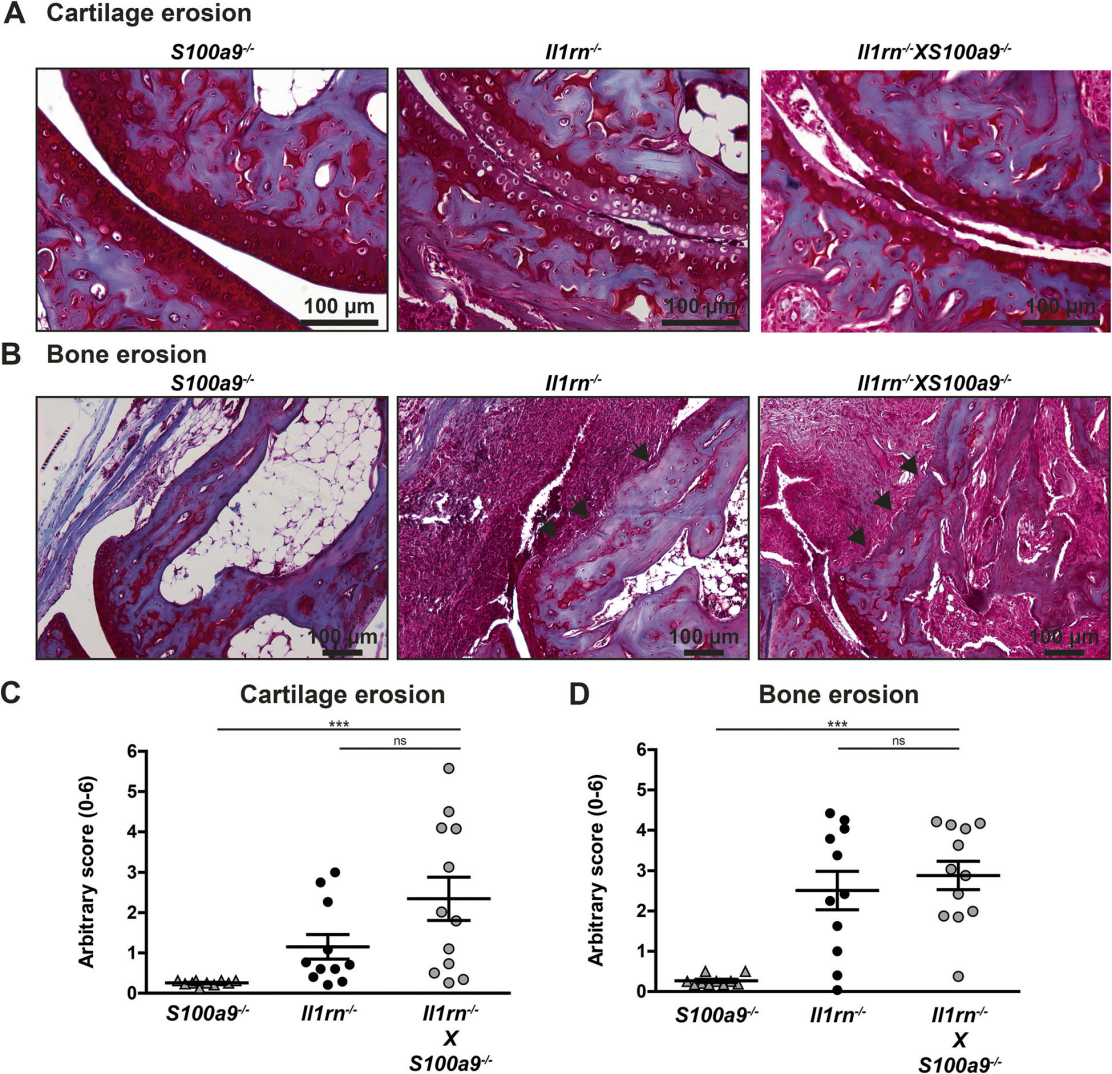
The absence of S100A9 does not ameliorate cartilage and bone erosion in 20-week-old *Il1rn*^*−/−*^ mice. Representative photomicrographs of Safranin-O/Fast Green-stained sections showing cartilage and bone erosion in the ankle joints of 20-week-old *S100a9*^*−/−*^, *Il1rn*^*−/−*^, and *Il1rn*^*−/−*^X*S100a9*^*−/−*^ mice (**A**, original magnification ×200 and **B**, original magnification ×100). Quantification of cartilage erosion at the tibia, proximal and distal sides of the talus, and proximal side of the navicular bone with an arbitrary score showed no significant differences between *Il1rn*^*−/−*^X*S100a9*^*−/−*^ and *Il1rn*^*−/−*^ mice (**C**). No significant differences in bone erosion were observed between *Il1rn*^*−/−*^X*S100a9*^*−/−*^ and *Il1rn*^*−/−*^ mice as quantified at 8 locations along the tibia, talus, and navicular bone (**D**). Scatterplots are shown, with horizontal and vertical lines representing mean ± SEM values. Each data point represents the sum score of the right and left ankle joints of one mouse. ns not significant, ***P < 0.001

Overall, although not significantly increased, 12-week-old *Il1rn*^*−/−*^X*S100a9*^*−/−*^ mice consistently showed a trend towards increased bone and cartilage erosion and showed increased numbers of pro-inflammatory Ly6C^high^ cells in the bone marrow and spleen. Therefore, we investigated whether joint pathology was affected by the absence of S100A8/A9 in *Il1rn*^*−/−*^ mice later after the onset of arthritis in 20-week-old mice. Severe cartilage and bone erosion were observed in the ankle joints of both *Il1rn*^*−/−*^ and *Il1rn*^*−/−*^X*S100a9*^*−/−*^ mice (Fig. 5A, B**)**. Quantification of cartilage and bone erosion confirmed a strong increase in joint damage in *Il1rn*^*−/−*^X*S100a9*^*−/−*^ compared to *S100a9*^*−/−*^ mice*.* Most severe cartilage and bone erosions were observed in the *Il1rn*^*−/−*^X*S100a9*^*−/−*^ mice in the distal parts of the talus and navicular bone, which we observed in 12-week-old mice as well but was more profound in this set of 20-week-old mice, although this did not result in a significantly higher average score as compared to *Il1rn*^*−/−*^ mice (Fig. 5C, D).

Additionally, although scoring of cartilage proteoglycan depletion might be strongly influenced by the presence of severe cartilage erosion, PG depletion was increased in *Il1rn*^*−/−*^X*S100a9*^*−/−*^ compared to *S100a9*^*−/−*^ mice. However, no differences were found between *Il1rn*^*−/−*^ and *Il1rn*^*−/−*^X*S100a9*^*−/−*^ mice (Additional file 4).

## Discussion

In the present study, we show that the absence of *S100a9* does not alter joint inflammation, cartilage destruction, and bone erosion in the ankle joints of *Il1rn*^*−/−*^ mice, suggesting that S100A8/A9 is not essential for the development of arthritis in the presence of such excessive IL-1 signaling.

We previously described that both local and serum levels of S100A8/A9 are strongly elevated in *Il1rn*^*−/−*^ mice with high IL-1 signaling and that these levels strongly correlated with inflammation and destruction parameters. Moreover, serum S100A8/A9 levels at a relatively early disease stage showed prognostic value for the development of inflammation later on. Together, these data show that S100A8/A9 may be crucially involved in the development of inflammation and pathology under these conditions of high IL-1 signaling [26]. Therefore, in the present study, we set out to investigate this possible involvement. However, we observed that the functional involvement of S100A8/A9 was very limited and not essential for the development of joint inflammation and destruction under these circumstances.

High levels of both the alarmin S100A8/A9 and the pro-inflammatory cytokine IL-1β are found in many patients suffering from various forms of arthritis. The involvement of these factors in the development of joint pathology has previously been demonstrated in other experimental models of RA. These studies showed that the absence of S100A8/A9 inhibited the development of joint pathology in antigen-induced arthritis (AIA), whereas the development of other models of experimental arthritis like the K/BxN serum transfer arthritis model and collagen-induced arthritis (CIA) was not affected, suggesting that the importance of S100A8/A9 is model-dependent [6, 28, 29]. On the other hand, whereas IL-1β was shown to be important for the development of CIA and K/BxN serum transfer arthritis [30–32], blocking this pro-inflammatory cytokine during AIA did not decrease the inflammation in this model [33]. Together, these data show that the dependency of the experimental arthritis models on S100A8/A9 or IL-1β is model-specific, which might be due to factors like the use of distinct triggers, local versus systemic inflammation, and variations in the predominant inflammatory cell types that are involved in the development of disease pathology in these models.

Normally, S100A8 and S100A9 monomers quickly form heterodimers in the cell. Once released upon cell stress, these heterodimers can activate the immune system predominantly via Toll-like receptor 4 (TLR4) and relatively quickly tetramerize under high calcium conditions, such as present in the extracellular environment, and therewith lose their capacity to activate the immune system in a TLR4-dependent manner. Genetic ablation of *S100a9* in mice does not only result in the loss of S100A9 expression, but also of functional S100A8 protein in the periphery because of the rapid degradation of the S100A8 monomers by the proteasome, although protein expression can be observed in the bone marrow of these mice [34, 35]. A recent study investigated the role of S100A8/A9 under conditions of high tumor necrosis factor α (TNFα), using mice deficient in tristetraprolin (*Ttp*^*−/−*^) and mice with a conditional overexpression of human TNFα (ih*TNFA*), which were additionally deficient in *S100a9*. Surprisingly, this resulted in a severe aggravation of inflammation and damage in the joints, which was attributed to a rescued expression of S100A8/A8 homodimers due to an inadequate breakdown of S100A8 by the proteasome [35]. S100A8/A8 homodimers lack the ability to tetramerize and therefore stay in a pro-inflammatory active state [35].

In the present study, we used *Il1rn*^*−/−*^ mice that lack the IL-1RA protein, which competitively blocks the binding of IL-1β to IL-1RI and thereby decreases IL-1 signaling [24]. Therefore, this mouse strain is often used as a model to study high IL-1 signaling [25]. Using immunohistochemical analysis, we confirmed that neither *S100a9*^*−/−*^ nor *Il1rn*^*−/−*^*XS100a9*^*−/−*^ mice showed S100A9 protein expression, whereas profound expression was observed in *Il1rn*^*−/−*^ mice (Additional file 5A). Nevertheless, in contrast to the earlier findings under circumstances of high TNFα, no rescue of S100A8 expression could be observed in the inflamed synovium of the ankle joints of *Il1rn*^*−/−*^*XS100a9*^*−/−*^, although it was clearly visible within the bone marrow, suggesting that, differently from TNFα, excessive IL1 signaling does not lead to accumulation of the S100A8 homodimer in inflammatory cells in the periphery. As expected, numerous S100A8-positive cells were present in the synovium and in the bone marrow of *Il1rn*^*−/−*^ mice (Additional file 5B). Elucidating the mechanisms underlying the discrepancy between these studies requires further investigation to reveal the complex interaction of TNFα, IL-1, and S100A8/A9 during RA, although it should be mentioned that the involvement of S100A8/A9 in these studies was investigated under very high TNFα and IL-1 signaling.

We did on the other hand, however, also not observe an amelioration of the pathology in the absence of *S100a9* as might be expected in the absence of a strong pro-inflammatory factor that has been shown to be involved in many experimental arthritis models. This could be the result of an already optimal activation of the IL-1 signaling in the absence of its natural regulator IL-1RA, which is sufficient to induce the joint pathology as seen in the *Il1rn*^*−/−*^ mice. TLR4, which is the most dominant receptor for S100A8/A9, and the IL-1β receptor IL-1R1 both activate the MyD88, IRAK, TRAF6 signaling pathway, and in this model, only IL-1 signaling in the absence of its natural inhibitor might already be sufficient to activate this intracellular signaling pathway to such an extent that the disease pathology progresses [36]. In agreement with this idea, it has been shown that the TLR4 ligand lipopolysachariden (LPS) is able to restore the development of K/BxN serum transfer arthritis in *Il1r1*^−/−^ mice, which were protected from the development of pathology in the absence of LPS [37]. A possible second mechanism to explain our findings is that, like S100A8/A9, other factors that are present during arthritis development can induce IL-1β expression and regulate its activation and as such be sufficient to fully activate IL-1β in *Il1rn*^*−/−*^*XS100a9*^*−/−*^ mice [38–41].

## Conclusion

In conclusion, our data further underline the model-specific effects of the alarmin S100A8/A9 and show that although S100A8/A9 is a good biomarker for the development of inflammation and cartilage and bone damage, it is not crucially involved in these processes in *Il1rn*^*−/−*^ mice.

## Supplementary Information

**Additional file 1:.** Scoring system for quantification of PG depletion, cartilage and bone erosion.

**Additional file 2:.** Gating strategy for flow cytometry analysis.

**Additional file 3:.** Seru`m S100A8/A9 levels correlate with inflammation, cartilage and bone erosion in the ankle joints.

**Additional file 4:.** Absence of S100A9 does not ameliorate proteoglycan depletion in 20-weeks-old Il1rn^-/-^ mice.

**Additional file 5:.** S100a9^-/-^XIl1rn^-/-^ do not show rescued expression of S100A8 in inflammatory cells within the arthritic joints.

## Data Availability

Data and materials will be available upon request. Since no large databases are formed within these experiments, the data will not be deposited in a repository.

## Abbreviations

αMEM: α-Minimum essential medium
AIA: Antigen-induced arthritis
APC: Allophycocyanin
APC-Cy7: Allophycocyanin-cyanine 7
CIA: Collagen-induced arthritis
DAMPs: Damage-associated molecular patterns
EDTA: Ethylenediaminetetraacetic acid
FCS: Fetal calf serum
FITC: Fluorescein isothiocyanate
IL: Interleukin
IL-1RI: Type 1 IL-1 receptor
IL-1RII: Type 2 IL-1 receptor
Il1rn: IL-1 receptor antagonist
LPS: Lipopolysachariden
M-CSF: Macrophage colony-stimulating factor
PE: Phycoerythrin
RANKL: Receptor activator of nuclear factor kappa-Β ligand
Rm: Recombinant mouse
TLR4: Toll-like receptor 4
TNF α: Tumor necrosis factor α
TRAP: Tartrate-resistant acid phosphatase
Ttp: Tristetraprolin
WT: Wild type
