# Supplementary material for: S100A8/A9 is not essential for the development of inflammation and joint pathology in interleukin-1 receptor antagonist knockout mice
Source: Arthritis Res Ther. 2021 Aug 19;23:216. doi: 10.1186/s13075-021-02602-y (PMC8375068; doi:10.1186/s13075-021-02602-y)
Supplement: Supplementary file 1 — Additional file 1:. Scoring system for quantification of PG depletion, cartilage and bone erosion. [file 13075_2021_2602_MOESM1_ESM.pdf]

Additional file 1

A

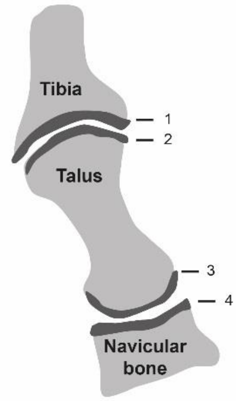

Cartilage erosion

SCORE 0	no erosion, no fibrillation, cartilage surface completely smooth
SCORE 0.5	fibrillation / irregular surface
SCORE 1	mild erosion of the non-calcified layer (25% of depth of non-calcified layer)
SCORE 1,5	severe erosion of non-calcified layer (50% of depth of non-calcified layer)
SCORE 2	erosion up until the tidemark
SCORE 2.5	erosion beyond the tidemark, part of the calcified cartilage layer still present
SCORE 3	complete erosion of calcified layer

PG depletion

SCORE 0	complete red staining of the superficial cartilage zone, equally stained as the deeper zone beyond the tidemark
SCORE 1	mild loss of red staining in the superficial cartilage zone, but still predominantly red
SCORE 2	moderate loss of red staining in the superficial cartilage zone, with the blue counterstaining starting to predominate throughout the remaining gloom of redness
SCORE 3	complete loss of red staining of the superficial cartilage zone, with only blue counterstaining remaining or absence of proteoglycans due to complete erosion of the non-calcified layer or complete erosion of non-calcified and calcified layer

B

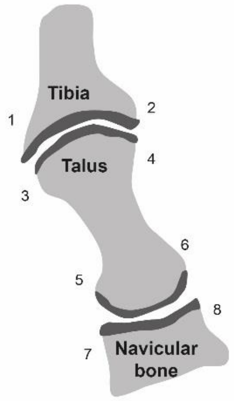

Bone erosion

SCORE 0	complete intact bone lining, no erosion
SCORE 1	superficial erosion
SCORE 2	deeper erosion, not yet extending to the bone marrow
SCORE 3	complete erosion of the bone structure, connecting the synovial cavity with the bone marrow cavity

Scoring system for quantification of cartilage erosion, cartilage proteoglycan depletion and bone erosion.

Cartilage erosion was evaluated using an arbitrary score ranging from 0 (no erosion) to 3 (complete erosion of the calcified cartilage layer) in four locations (1- tibia, 2-upper talus, 3-bottom talus, 4-upper navicular bone). Proteoglycan depletion in the articular cartilage was evaluated in the same four locations as cartilage erosion and scored on a scale from 0 (no PG depletion) to 3 (complete PG depletion). In case of a complete erosion of the non-calcified layer or complete erosion of non-calcified and calcified layer, PG depletion was scored as 3, since proteoglycans are completely absent under these conditions. The score of the four locations was averaged **(A).** Bone erosion was evaluated with an arbitrary score ranging from 0 (no erosion) to 3 (connection between joint cavity and bone marrow or more extensive bone destruction) in eight locations and the score of the eight locations was averaged **(B)**.
