# Supplementary material for: S100A8/A9 is not essential for the development of inflammation and joint pathology in interleukin-1 receptor antagonist knockout mice
Source: Arthritis Res Ther. 2021 Aug 19;23:216. doi: 10.1186/s13075-021-02602-y (PMC8375068; doi:10.1186/s13075-021-02602-y)
Supplement: Supplementary file 2 — Additional file 2:. Gating strategy for flow cytometry analysis. [file 13075_2021_2602_MOESM2_ESM.pdf]

Additional File 2

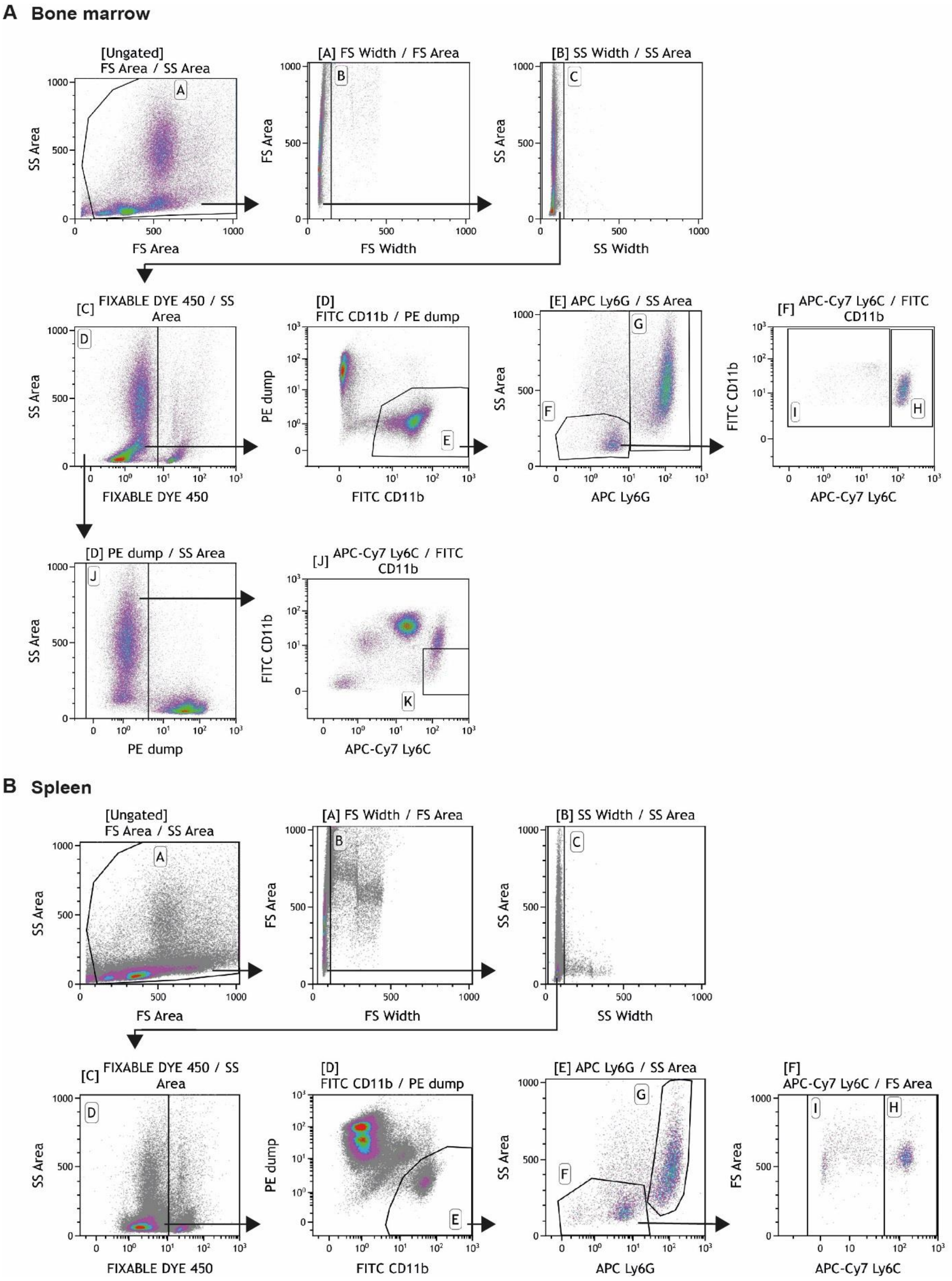

Gating strategy for flow cytometry analysis.

Gating strategy for flow cytometry analysis used to identify various myeloid populations in the bone marrow **(A)** and spleen **(B)** of mice. First, debris was excluded **(gate A)** and single and live cells were selected **(gate D)**. Subsequently, cells negative for Ter-119, CD3, CD45R/B220, CD49b, NK1.1 and positive for CD11b were selected (myeloid cells) **(gate E)**. Within the myeloid cell population, neutrophils (Ly6G^{high} and SSC$^{high/int}$) **(gate G)** and monocytes (Ly6G^{neg} and SSC^{low}) **(gate F)** were identified. Monocytes were further subdivided into the Ly6c^{low} and Ly6C^{high} subpopulations **(gate I** and **H** respectively**)**. Additionally, in the bone marrow the osteoclast precursor population Ly6C^{high}CD11b$^{low/neg}$ was identified; after exclusion of Ter 119, CD3, CD45R/B220, CD49b, NK1.1 positive cells **(gate J)**, cells were gated for their expression of CD11b and Ly6C (CD11b$^{low/neg}$ Ly6C^{high}) **(gate K)**.
