# Supplementary material for: S100A8/A9 is not essential for the development of inflammation and joint pathology in interleukin-1 receptor antagonist knockout mice
Source: Arthritis Res Ther. 2021 Aug 19;23:216. doi: 10.1186/s13075-021-02602-y (PMC8375068; doi:10.1186/s13075-021-02602-y)
Supplement: Supplementary file 3 — Additional file 3:. Seru`m S100A8/A9 levels correlate with inflammation, cartilage and bone erosion in the ankle joints. [file 13075_2021_2602_MOESM3_ESM.pdf]

**Additional File 3**

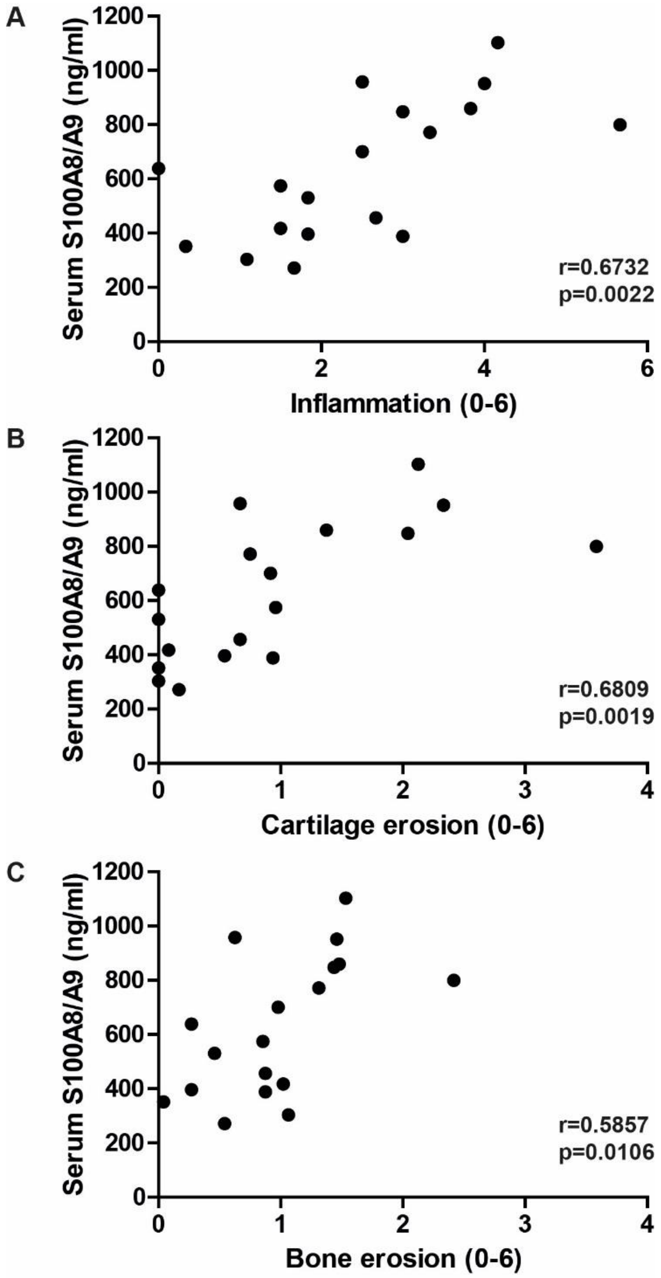


***Serum S100A8/A9 levels correlate with inflammation, cartilage and bone erosion in the ankle joints.***

Serum S100A8/A9 concentrations were determined by an in house developed sandwich ELISA as described previously (Vogl T *at al.* Nat Commun. 2014;5:4593, DOI: 10.1038/ncomms5593). Serum S100A8/A9 levels positively and significantly correlated with the severity of inflammation **(A),** cartilage erosion **(B)** and bone erosion **(C)** in the ankle joints of 12 weeks-old *Il1rn*$^{-/-}$ mice. r = Spearman's rank correlation coefficient.
