# Supplementary material for: S100A8/A9 is not essential for the development of inflammation and joint pathology in interleukin-1 receptor antagonist knockout mice
Source: Arthritis Res Ther. 2021 Aug 19;23:216. doi: 10.1186/s13075-021-02602-y (PMC8375068; doi:10.1186/s13075-021-02602-y)
Supplement: Supplementary file 4 — Additional file 4:. Absence of S100A9 does not ameliorate proteoglycan depletion in 20-weeks-old Il1rn-/- mice. [file 13075_2021_2602_MOESM4_ESM.pdf]

Additional File 4

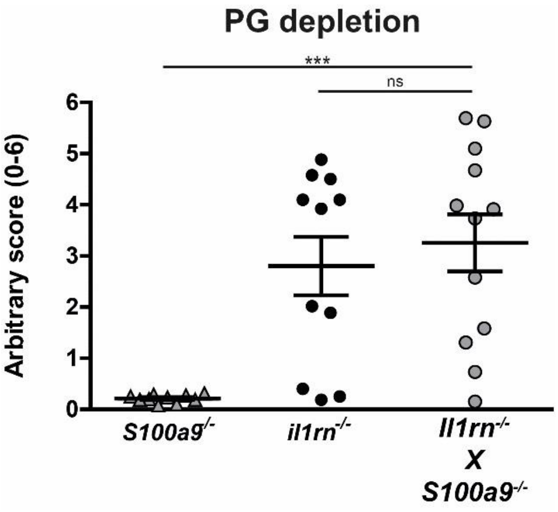

Absence of* S100a9 *does not ameliorate proteoglycan depletion in 20-weeks-old Il1rn$^{-/-}$ mice. Quantification of proteoglycan depletion at the tibia, proximal and distal side of the talus and the proximal side of the navicular bone with an arbitrary score showed an increase in *Il1rn$^{-/-}$*X*S100a9$^{-/-}$* compared to *S100a9$^{-/-}$* mice whereas no significant differences were observed between *Il1rn$^{-/-}$* X*S100a9$^{-/-}$* and *Il1rn$^{-/-}$* mice. It is, however, important to consider that a high score of PG depletion might be a reflection of the cartilage erosion rather than proteoglycan depletion per se, since complete erosion of the cartilage result in absence of proteoglycans and for that reason was given the highest score. Scatterplots are shown, with horizontal and vertical lines representing mean ± SEM values. Each data point represents the sum score of the right and left ankle joints of one mouse. ns = not significant, *** $P < 0.001$).

.
