# Supplementary material for: S100A8/A9 is not essential for the development of inflammation and joint pathology in interleukin-1 receptor antagonist knockout mice
Source: Arthritis Res Ther. 2021 Aug 19;23:216. doi: 10.1186/s13075-021-02602-y (PMC8375068; doi:10.1186/s13075-021-02602-y)
Supplement: Supplementary file 5 — Additional file 5:. S100a9-/-XIl1rn-/- do not show rescued expression of S100A8 in inflammatory cells within the arthritic joints. [file 13075_2021_2602_MOESM5_ESM.pdf]

**Additional File 5**

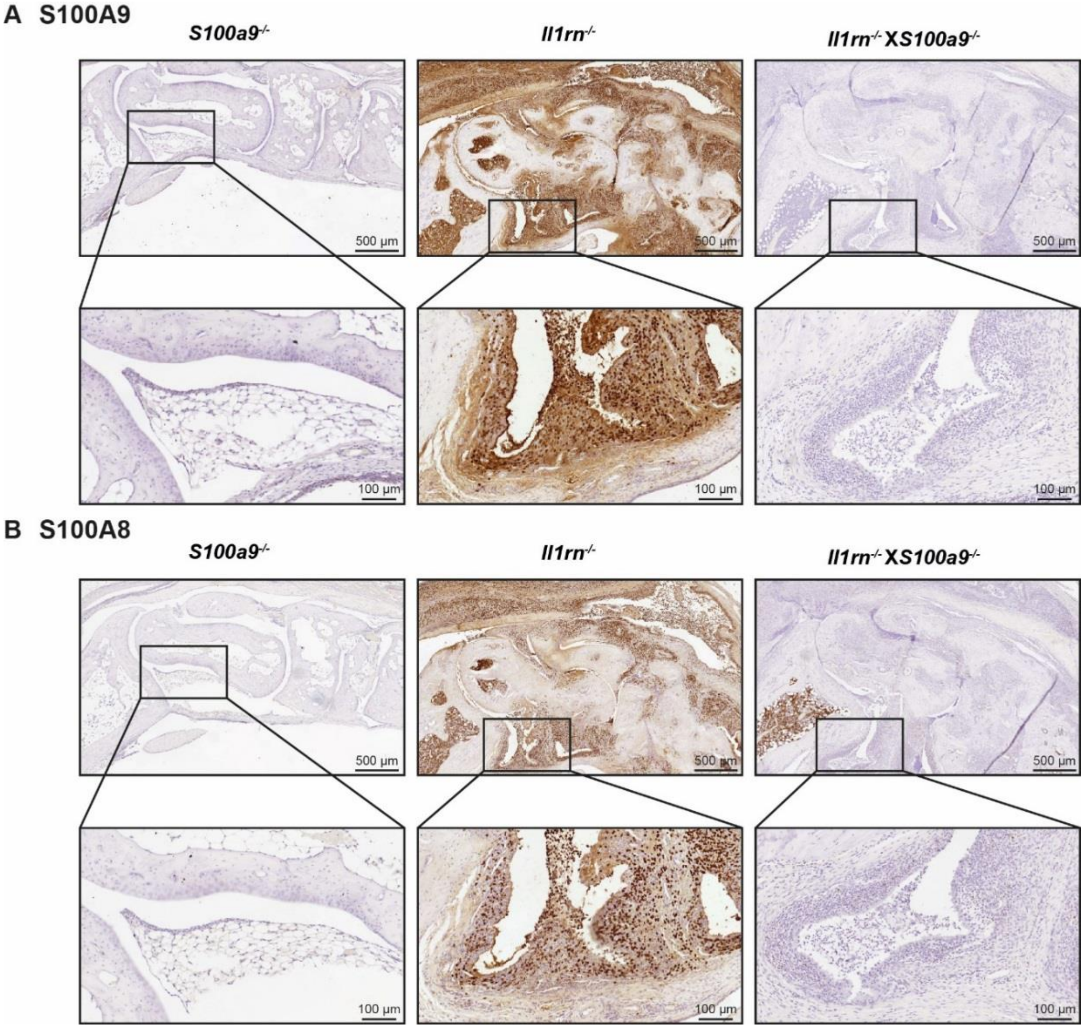


***S100a9*$^{-/-}$*XIl1rn*$^{-/-}$ *do not show rescued expression of S100A8 in inflammatory cells within the arthritic joints.***

The expression of S100A8 and A9 in the ankle joint sections of 20-weeks-old *S100a9*$^{-/-}$*, Il1rn*$^{-/-}$ and *Il1rn*$^{-/-}$X*S100a9*$^{-/-}$ mice was determined with immunohistochemistry. Hereto, sections were digested with proteinase-free chondroitinase ABC (0.25 units/ml in 0.1 M Tris-HCl, pH 8.0; Sigma-Aldrich) for antigen retrieval followed by overnight incubation with rabbit anti-S100A8 or anti-S100A9 (*own facilities*). Sections were then incubated with biotinylated goat anti-rabbit IgG (Dako) as a secondary antibody followed by incubation with avidin-streptavidin-peroxidase (Elite-kit, Vector). Antibody binding was visualized using 3,3′-diaminobenzidine (DAB; Powervision DAB, Immunologic) and sections were counterstained with hematoxylin. Representative photomicrographs of S100A9 **(A)** and S100A8 **(B)** immunohistochemical staining are shown. S100A9 positive cells were absent in bone marrow and in synovium of *S100a9*$^{-/-}$ and of *Il1rn*$^{-/-}$X*S100a9*$^{-/-}$ mice, whereas numerous *S100A9* positive cells were visible in bone marrow (BM) and inflamed ankles of Il*1rn*$^{-/-}$ mice. High numbers of S100A8 positive cells were present in the ankle joints of *Il1rn*$^{-/-}$ mice. Note that expression of S100A8 protein is visible in BM of *Il1rn*$^{-/-}$X*S100a9*$^{-/-}$ mice but not in the inflamed synovium suggesting that S100A8 expression is not rescued in the periphery under circumstances of high IL-1 signaling.
